# Evaluation of the ThroLy score for the prediction of venous thromboembolism in newly diagnosed patients treated for lymphoid malignancies in clinical practice

**DOI:** 10.1002/cam4.1540

**Published:** 2018-05-15

**Authors:** Joanna Rupa‐Matysek, Katarzyna Brzeźniakiewicz‐Janus, Lidia Gil, Zbigniew Krasiński, Mieczysław Komarnicki

**Affiliations:** ^1^ Department of Hematology and Bone Marrow Transplantation Poznan University of Medical Sciences Poznań Poland; ^2^ Department of Hematology Multi‐Specialist Hospital, Gorzow Wlk. University of Zielona Gora Gorzow Wielkopolski Poland; ^3^ Department of General and Vascular Surgery Poznan University of Medical Sciences Poznań Poland

**Keywords:** diffuse large B‐cell lymphoma, Hodgkin lymphoma, ThroLy risk score, venous thromboembolism, venous thromboembolism risk assessment model

## Abstract

The utility in clinical practice of a recently developed and validated predictive model for venous thromboembolism (VTE) events in lymphoma patients, known as the thrombosis lymphoma (ThroLy) score, is unknown. We evaluated the association of ThroLy with VTE in patients treated for diffuse large B‐cell lymphoma (DLBCL) or Hodgkin lymphoma (HL) undergoing ambulatory first‐line chemotherapy. Retrospective analyses were performed on 428 patients (median age 50), 241 were newly diagnosed DLBCL, and 187 had HL. During initial chemotherapy, 64 (15%) patients developed VTE. According to the ThroLy, 322 (75.2%) patients were considered low risk, 88 (20.6%) patients had intermediate risk and 18 (4.2%) patients high risk for VTE development. Patients with DLBCL were more often in the high‐risk ThroLy group and had more VTE events than HL. VTE occurred in; 38.9% (n = 7) high‐risk patients, 29.5% (n = 26) intermediate risk, and 9.6% (n = 31) low risk according to the ThroLy score. However, in multivariate analysis, high ThroLy (OR 5.13; 95% CI: 1.83‐14.36, *P* = .002), intermediate ThroLy (OR 3.96; 95% CI: 2.19‐7.17, *P *<* *.001), and aggressive lymphoma‐DLBCL (OR 1.91; 95% CI: 1.05‐3.47, *P *=* *.034) were all significantly associated with development of VTE, 48% of the VTE events occurred in the low‐risk ThroLy score group (the ROC AUC (95% CI) 0.40‐0.70 and C statistic‐0.55). In our study, the ThroLy score was not a suitably accurate model for predicting VTE events in patients at higher risk of VTE. Further research should be conducted to identify new biomarkers that will predict these events and to establish a new VTE risk assessment model.

## 1 INTRODUCTION

Patients with cancer are at a high risk of venous thromboembolism (VTE), which increases mortality and causes deterioration in the quality of life.^1^, ^2^ Due to the heterogeneity of cancer and the various risks of VTE development including different biomarkers,^3^ currently no routine prophylaxis is recommended in any guidelines for outpatients with cancer receiving chemotherapy with a few exceptions.^4^, ^5^, ^6^, ^7^ At present, several VTE‐assessment models for chemotherapy‐associated thrombosis exist, which are intended to help in the identification of patients at a higher risk of VTE 8, 9, 10, 11, 12 and who may possibly benefit from thromboprophylaxis. Due to poor discriminatory performance, most of the recently established VTE‐assessment models have proven to be of limited clinical utility because of the low predictive value of VTE events in subsequent studies, particularly in studies based on a single cancer cohort.^13^, ^14^, ^15^, ^16^

It is believed that the risk to patients with lymphoma, undergoing ambulatory chemotherapy, of developing VTE is similar to that of patients with solid tumours with the incidence reaching 14.6%.^17^, ^18^ Lymphoma is considered to constitute a high risk of VTE development in the best‐validated model to stratify outpatients with cancer, which was developed by Khorana and is known as the Khorana risk score (KRS).^8^ Based on these data and some variables from the KRS, Antic et al developed and validated a multivariable model for thromboembolic events in lymphoma patients known as the Thrombosis Lymphoma (ThroLy) score.^19^ To this day, there have been no external validation studies to evaluate the ThroLy score in clinical practice.^20^

Thus, the aim of this study was to determine the discriminatory performance of the ThroLy score in stratifying or predicting VTE events in patients treated for newly diagnosed diffuse large B‐cell lymphoma (DLBCL) and Hodgkin lymphoma (HL). The second goal of our study was to compare the performance of the ThroLy score between two different histologic subtypes of lymphoma, DLBCL and HL, respectively.

## 2 METHODS

To assess the occurrence of VTE, newly diagnosed patients with HL or DLBCL receiving first‐line chemotherapy in one hematological centre were retrospectively analyzed. The ECOG/WHO performance status of most analyzed patients was 0‐2 and the patients received chemotherapy (ABVD for HL and CHOP‐R for DLBCL) in the outpatient clinic of the Department of Haematology and Bone Marrow Transplantation at Poznan University of Medical Sciences between June 2009 and July 2016. Disease progression, occurrence of VTE or death, or the end date of the study (December 2016) defined the observation time.

To evaluate whether the ThroLy score discriminates between patients with low or high risk of thrombotic events in our patients with lymphoma, patients who had received therapeutic (full‐dose) anticoagulation (low molecular weight heparin, vitamin K antagonist or direct oral anticoagulants) due to acute VTE events or atrial fibrillation at the start of chemotherapy were excluded from the study. Neither erythropoiesis‐stimulating agents nor implantation of central venous catheter were recorded during their first‐line therapy.

There was no routine screening for VTE. Ultrasounds with Doppler and color imaging were used to diagnose deep vein thrombosis (DVT) only in symptomatic patients and (spiral) computed tomography angiography (CTA) was performed to detect pulmonary embolism (PE). Most patients had initial positron‐emission tomography with computed tomography (PET‐CT) to evaluate the stage of lymphoma.

All patients’ clinical and laboratory data including the presence of systemic symptoms, mediastinal bulky involvement defined as the longest measurement of a tumor mass of 10 cm or greater, stage of disease according to the Lugano classification, International Prognostic Score (IPS) for HL, International Prognostic Index (IPI) score for DLBCL and ThroLy score were collected prior to chemotherapy.^19^, ^21^, ^22^, ^23^, ^24^

The patients were categorized into low (0‐1 point), intermediate (2‐3 points), and high‐risk categories (>3 points) using the ThroLy model, based on previous VTE/acute myocardial infarction/stroke (2 points), reduced mobility (ECOG 2‐4, 1 point), obesity (BMI > 30 kg/m^2^, 2 points), extranodal localization (1 point), mediastinal involvement (2 points), neutrophils <1 × 10^9^/L (1 point), and hemoglobin level <100 g/L (1 point), Table 1.^19^ For the ThroLy model, a full blood count was performed by standard methods.

**Table 1:** Predictive model for thromboembolism according to the ThroLy model developed by Antic et al^19^

Patient characteristics	Assigned score
Previous VTE/acute myocardial infarction/stroke	2
Reduced mobility (ECOG 2‐4),	1
Obesity (BMI > 30 kg/m^2^)	2
Extranodal localization	1
Mediastinal involvement	2
Neutrophils <1 × 10^9^/L	1
Hemoglobin level <100 g/L	1

ThroLy score points: 0‐1, low risk; 2‐3, intermediate risk and score >3, high risk.

The Bioethical Committee of Poznan University of Medical Sciences approved the study, in accordance with the Declaration of Helsinki (No KB‐1028/17).

### 2.1 Statistical analysis

Assuming a VTE event rate of about 7%‐14.6% based on averages from literature,^25^, ^26^, ^27^ we calculated that at least 91‐164 patients would be required to determine the role of the ThroLy score with a power of 95% using a two‐side test at an alpha level of 0.05 when the size of the population is small, <1000. Descriptive statistics, such as the frequency (n), arithmetic mean ($\bar{x}$), and standard deviation (SD), are presented for normally distributed variables. Otherwise, medians and the standard errors (SE) with interquartile ranges (25 and 75 percentile) were used. The Shapiro‐Wilk test was performed to assess normality. To compare differences between the groups, the chi‐square test was used for categorical variables and the Mann‐Whitney *U*‐test for continuous variables.

Univariate logistic regression was used to evaluate potential risk factors that may influence VTE. A multivariate analysis was performed with selected variables that were significant in the univariate analysis *(P *<* *.01). In each model, the odds ratio (OR) for each independent variable was determined with a confidence interval (CI) of 95%.

Receiver operating characteristic (ROC) curve analysis was performed to determine the area under the curve (ROC AUC, C statistic) values predictive of VTE development for the evaluation of overall population, HL group, and DLBCL group. We also calculated the sensitivity (probability of high risk in those patients experiencing VTE), specificity (probability of high risk in those not experiencing VTE), and determined ROC AUC with a confidence interval (CI) of 95% for VTE development.

The probabilities of VTE‐free survival were estimated via the Kaplan‐Meier method, and the comparisons were performed using the chi‐square test. A *P*‐value below .05 was regarded as statistically significant. The statistical analyses were performed with STATISTICA 13 and STATISTICA Medical Package 2.0 (StatSoft, Inc., Tulsa, Oklahoma, USA).

## 3 RESULTS

Four hundred and twenty‐eight adult patients were included in the study; 241 patients with diagnosed DLBCL and 187 with HL. The median age was 50 years (range 18‐98 years), of whom 51% were females. The median observation time was 37 months (range 0.5‐92).

Although 69% of patients were presented with advanced lymphoma (n = 297, stage IV), only 42% of cases (n = 178) were classified as having a high‐risk disease (IPI ≥ 3 or IPS ≥ 3).

Bulky disease with mediastinal involvement was more often observed in patients with HL than the DLBCL group (*P *=* *.008). Except for the older age and more advanced stage category of the DLBCL group (*P *<* *.001), there were no significant differences in gender distribution, presence of systemic symptoms or incidence of a high‐risk disease between the HL and DLBCL patients (Table 2). Among the patients with DLBCL, there were only three obese patients (BMI > 30 kg/m^2^) and 13 patients started the treatment with ECOG 2. None of the patients with HL had obesity (BMI > 30 kg/m^2^) nor reduced mobility (ECOG 2‐4). Only 14 patients had neutrophils below 1 × 10^9^/L at diagnosis. Thirty‐four (8%) patients had previous VTE/acute myocardial infarction/stroke, and most of them (n = 33) had received only prophylactic aspirin at the time of diagnosis.

**Table 2:** Patients’ characteristics

Characteristic	Overall populationn = 428	DLBCL^a^n = 241	HL^a^n = 187	*P* value
Median age, range years	50 (18‐98)	60 (18‐98)	36 (18‐84)	<.0001
Sex, male n (%)	209	123 (51%)	86 (46%)	.3000
Advanced disease^b^	218	158 (66%)	60 (32%)	<.0001
Extranodal localization	199	140 (58%)	59 (32%)	<.0001
Systemic symptoms	258	146 (61%)	112 (60%)	.8853
Mediastinal involvement	45	17 (7%)	28 (15%)	.0081
High‐risk disease^c^	178	105 (44%)	73 (39%)	.3455
Previous VTE/AMI/stroke	34	32 (13%)	2 (1%)	<.0001
Hemoglobin level <100 g/L	44	23 (10%)	21 (11%)	.4688
Neutrophils <1 × 10^9^/L	14	10 (4%)	4 (2%)	.2462
High ThroLy score^e^	18	15 (6%)	3 (2%)	.0576
Intermediate ThroLy score^d^	88	50 (21%)	38 (20%)	
Presence of VTE	64	45 (19%)	19 (10%)	.0143

a The percentages are related to the numbers given in the first column of the same line.

b Advanced disease: stage according to Lugano IV.

c International Prognostic Index ≥3; International Prognostic Score ≥3.

According to the ThroLy score; high risk (Score > 3).

d According to the ThroLy score; intermediate risk (Score 2 – 3).

*P *<* *.05‐statistically significant.

Overall, 64 (15%) patients developed venous thromboembolism in the median 4.7 months (25th‐75th percentile: 1.4‐7.6), of whom 45 were patients with DLBCL and 19 were cases with HL (19% vs 10%, *P *=* *.0143). Symptomatic pulmonary embolism was diagnosed in 11% of these patients (7/64) with VTE, deep vein thrombosis of lower extremities was found in 28% of cases (18/64), and other site deep vein thrombosis including internal jugular vein (n = 23), portal vein (n = 1), and upper extremity thrombosis (n = 15) were detected in 61% of the patients who developed VTE (39/64). Patients with mediastinal involvement had VTE events more often than patients without (26% vs 8%, *P *<* *.0001) (Table 3). Despite the fact that the patients with previous VTE/acute myocardial infarction/stroke received prophylactic aspirin, 11 of 34 patients developed VTE (*P *=* *.0030, Table 3).

**Table 3:** Comparison of patients’ characteristics with/or without VTE

	Overall population n = 428	VTE group during follow‐up^a^ n = 64	Non‐VTE group during follow‐up^a^ n = 364	*P* value
Median age, range years	50 (18‐98)	49 (22‐81)	50 (18‐98)	.9698
Sex, male n (%)	209 (49%)	34 (53%)	175 (48%)	.4562
Aggressive lymphoma: DLBCL	241 (56%)	45 (70%)	196 (54%)	.0143
Advanced disease^b^	218 (51%)	38 (59%)	180 (49%)	.1430
Extranodal localization	199 (46%)	37 (58%)	162 (44%)	.0490
Systemic symptoms	258 (60%)	23 (36%)	41 (64%)	.5025
Mediastinal involvement	45 (11%)	17 (26%)	28 (8%)	<.0001
High‐risk disease^c^	178 (42%)	34 (53%)	144 (40%)	.0423
Previous VTE/AMI/stroke	34 (8%)	11 (17%)	23 (6%)	.0030
Reduced mobility (ECOG 2‐4)	13 (3%)	5 (8%)	8 (2%)	.0158
Hemoglobin level <100 g/L	44 (10%)	11 (17%)	33 (9%)	.0485
Neutrophils <1 × 10^9^/L	14 (3%)	2 (3%)	12 (3%)	.9432
High ThroLy score^d^	18 (4%)	7 (11%)	11 (3%)	<.0001
Intermediate ThroLy score^d^	88 (21%)	26 (41%)	62 (17%)	

a The percentages are related to the numbers given in the first column of the same line.

b Advanced disease: stage according to Lugano IV.

c IPI, International Prognostic Index ≥3; IPS, International Prognostic Score ≥3.

d According to the ThroLy score; high risk (Score > 3).

According to the ThroLy score; intermediate risk (Score 2 – 3).

*P *<* *.05‐statistically significant.

During a median follow‐up of 37 months (range 0.5‐92), 56 patients (13%) died, including 16 patients from the group with VTE and 39 patients from the group without VTE. No impact of a high ThroLy on prognosis was found (chi‐square test = 1.18, *P *=* *.5544).

According to the ThroLy score, 18 (4%) patients were considered to be at high risk, 88 (21%) to be at intermediate risk and 322 (75%) to be at low risk of thrombosis development. VTE occurred in 39% (7/18) of the high‐risk patients and in 29% (26/88) of the intermediate risk and in 10% (31/322) of the low‐risk patients according to ThroLy. The high‐risk and the intermediate‐risk ThroLy patients were more often diagnosed with DLBCL than HL (*P *=* *.0576). Among the low VTE risk ThroLy category (n = 322), 31 patients developed VTE which comprised 48% (31/64) of all VTE cases in the studied population (*P *<* *.0001). Most of the low‐risk patients with VTE were treated for HL (19/31, 61%).

**Figure 1:**
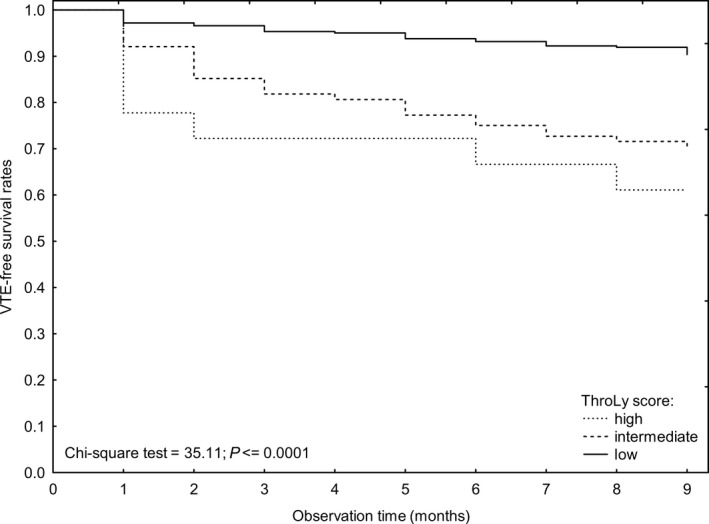
Kaplan‐Meier analysis of VTE‐free survival rates according to the ThroLy category (high, intermediate, and low risk)

In a Kaplan‐Meier analysis of VTE‐free survival rates, significant differences were found between the patients in a high ThroLy category and those patients in a low or intermediate ThroLy group (chi‐square test = 35.11, *P *<* *.001), Figure 1.

At the cutoff point for the high‐risk category (score ≥3), we calculated the sensitivity (probability of high risk in those patients experiencing VTE), specificity (probability of high risk in those not experiencing VTE), and determined ROC AUC with a confidence interval (CI) of 95% for VTE development. For HL, the sensitivity was 76%, the specificity 33% and the ROC AUC (95% CI) 0.20‐0.90. For DLBCL, the sensitivity was 66%, the specificity 40%, and the ROC AUC (95% CI) 0.36‐0.70 (Table 4). For all subjects, the sensitivity was 61%, the specificity 29%, and the ROC AUC (95% CI) 0.40‐0.70. The C statistic was 0.53 for the DLBCL group, 0.55 for the HL group, and 0.55 for the entire group. In our patients treated for DLBCL and HL, the ThroLy score discriminated poorly between patients with high and low/intermediate risk of VTE development.

**Table 4:** VTE rates and negative and positive predictive value of development of VTE from the ThroLy Score in the studied lymphoma patients

Risk group	Patients n	VTE n	AUC (95% CI)	C statistic
HL				
Low/intermediate	38	9	0.20‐0.90	0.55
High	3	1		
DLBCL				
Low/intermediate	50	17	0.36‐0.70	0.53
High	15	6		
Overall population				
Low/intermediate	88	26	0.40‐0.70	0.55
High	18	7		

AUC indicates area under the curve; CI indicates confidence interval.

### Factors associated with VTE

Aggressive lymphoma (DLBCL), high‐risk disease, bulky disease with mediastinal involvement, previous VTE/AMI/stroke, reduced mobility, prechemotherapy leukocyte count >11 × 10^9^/L, high and intermediate ThroLy score were all significantly associated with an increased risk of VTE by univariate analysis (Table 5). Patients with advanced disease and pre‐chemotherapy hemoglobin below 10 g/dL had only a trend toward an increased risk of VTE. In the multivariate analysis, a high ThroLy score (OR 5.13; 95% CI: 1.83‐14.36, *P *=* *.002), an intermediate ThroLy score (OR 3.96; 95% CI: 2.19‐7.17, *P *<* *.001), and aggressive lymphoma‐DLBCL (OR 1.11; 95% CI: 1.05‐3.47, *P *=* *.034) remained significant for VTE development (Table 6).

**Table 5:** Univariate analyses of determining factors that affect VTE development in patients with lymphoid malignancies

Factor	Univariate analysis	
	Odds Ratio (95% CI)	*P* value
Sex (male)	0.65 (0.38‐1.12)	.1208
Age	1.00 (0.98‐1.11)	.9610
Aggressive lymphoma: DLBCL	2.03 (1.14‐3.61)	.0157
Advanced disease^a^	1.61 (0.94‐2.77)	.0844
Extranodal localization	1.71 (0.99‐2.92)	.0507
Systemic symptoms	0.90 (0.52‐1.55)	.6940
Mediastinal involvement	4.34 (2.21‐8.53)	.0001
High‐risk disease^b^	0.90 (0.52‐1.55)	.6940
Previous VTE/AMI/stroke	3.08 (1.42‐6.68)	.0045
Reduced mobility (ECOG 2‐4)	3.77 (1.19‐11.92)	.0238
Neutrophils <1 × 10^9^/L	0.95 (0.21‐4.33)	.9432
Prechemotherapy platelet count >350 × 10^9^/L	1.15 (0.63‐2.12)	.6495
Prechemotherapy leukocyte count >11 × 10^9^/L	1.81 (1.01‐3.26)	.0474
Prechemotherapy hemoglobin <100 g/L	0.48 (0.23‐1.01)	.0526
High ThroLy score^c^	5.97 (2.16‐16.52)	.0006
Intermediate ThroLy score^c^	3.94 (2.18‐7.09)	.0001

a Advanced disease: stage according to Lugano IV.

b IPI, International Prognostic Index ≥3; IPS, International Prognostic Score ≥3.

According to the ThroLy score; high risk (Score > 3).

c According to the ThroLy score; intermediate risk (Score 2 – 3).

*P *<* *.05‐statistically significant.

CI, confidence interval.

**Table 6:** Multivariate analyses determining factors that affected VTE development in patients with lymphoid malignancies

Variable	β	OR	95% CI	*P* value
Intercept	−2.634			
High ThroLy Score	1.635	5.13	1.83‐14.36	.002
Intermediate ThroLy Score	1.376	3.96	2.19‐7.17	<.001
Aggressive lymphoma: DLBCL	0.647	1.91	1.05‐3.47	.034

CI, confidence interval; OR, Odds ratio.

## 4 DISCUSSION

To our knowledge, this is the first external analysis that has evaluated the utility of the ThroLy score in stratifying or predicting VTE events in patients treated for newly diagnosed DLBCL and HL. The study also included a comparison between the two histologic subtypes of lymphoma with regard to the ThroLy score.

The reported incidence rate of VTE in patients with lymphoid malignancies undergoing ambulatory chemotherapy is as high as 14.6%.^17^, ^18^ This is consistent with our study in which very similar data were obtained at the level of 15.1%. The risk of VTE varies between patients due to general, patient‐related, disease‐related, and treatment‐related risk factors.^28^, ^29^ Furthermore, there are significant differences in VTE risk between patients undergoing treatment for various histological subtypes of lymphoma (indolent vs aggressive, previously classified as low‐grade vs high‐grade lymphoma) and different localizations, there is an especially high risk in the case of central nervous system involvement. Despite the relatively high incidence of VTE, routine pharmacological thromboprophylaxis is not recommended in most international guidelines 4, 5, 6, 7 and there are also limited data supporting the clinical benefit of thromboprophylaxis due to the unclear risk of bleeding.^30^, ^31^, ^32^, ^33^, ^34^, ^35^ There are ongoing studies aiming to identify patients with cancer and a higher risk of VTE development based on clinical and laboratory markers. Several reports have studied, developed, and validated VTE‐assessment models in order to predict VTE events in ambulatory patients undergoing treatment for cancer, including the best‐validated Khorana Risk Score (KRS).^8^, ^9^, ^10^, ^11^, ^12^ Recently research shows that the KRS failed to discriminate between high‐ and low‐risk patients with cancer for VTE development, including patients with lymphoma.^13^, ^14^, ^15^, ^16^, ^36^, ^37^ This is further supported by our study in which the KRS did not adequately predict VTE events in patients at a higher risk of VTE in a cohort of patients with lymphoid malignancies.^37^ Therefore, Antic et al developed the prognostic Thrombosis Lymphoma (ThroLy) score which is more specific for lymphoma patients than any other available scores targeting thrombosis in cancer patients.^19^ Although the ThroLy model has been evaluated in both an initial derivation and a validation cohort, it has not been externally evaluated yet.

In our cohort of patients with lymphoid malignancies, we were not able to provide enough evidence of the utility of the ThroLy score in the prediction of VTE events. In both the entire cohort and in each lymphoma histologic subtype, the C statistic was on average 0.55, much less than in the derivation and validation cohorts of the ThroLy model (C statistic >0.85). There might be several reasons for these differences. First of all, both venous and arterial thrombotic events in lymphoma patients were evaluated in the development and validation of the ThroLy score. In the ThroLy study, one‐quarter of thromboembolic events were arterial thromboembolisms such as strokes or acute myocardial infarctions. However, we analyzed only the relation between the risk‐group of the ThroLy score and the occurrence of VTE within first‐line chemotherapy. Besides, considering the younger age of the HL patients with no or few risk factors for cardiovascular disease or stroke, arterial thromboembolisms are less likely to happen in this group. Secondly, although, in both the univariate and multivariate logistic regression models, patients in the intermediate‐ and high‐risk ThroLy groups have nearly threefold to fivefold increased risk of VTE development, nearly half of the VTE events (48%) were detected in patients with a low‐risk ThroLy score, irrespective of the analyzed histologic lymphoma subtype. Detailed analyses of each variable included in the full multivariable prognostic logistic model of the ThroLy score were performed showing the lack of utility of several variables in the present study. Most patients reported constitutional symptoms (60%), including weight loss; the obesity value has limited application because only three patients in the DLBCL group and no patients in the HL group were obese (BMI >30 kg/m^2^). The percentage of obesity was also low in the ThroLy study (1.2%‐1.7%). Similar to the ThroLy cohort, patients with poorer performance status constituted approximately 2.5%‐3% of the population (ECOG ≥2). Due to the fact that one‐third of the patients with a previous history of VTE/AMI/stroke (8% of the study population) developed VTE, these variables should be considered as high risk for VTE events. In line with the ThroLy study, we showed the influence of a low prechemotherapy hemoglobin level. This was contrary to the findings of Posch et al who did not find any impact of low prechemotherapy hemoglobin levels in cancer patients on cancer‐associated thrombosis.^38^ Consistent with the ThroLy results and literature,^19^, ^39^ we confirmed the impact of the presence of mediastinum involvement on VTE risk. In our study population, we were unable to show any significance of extranodal localization or the presence of neutropenia (<1 × 10^9^/L) on the VTE risk. Thus, the risk of VTE events is the highest at the beginning of treatment; the VTE risk assessment was performed before initiation of therapy. In the present study, prechemotherapy neutropenia was found in only 3% of all patients. Moreover, patients with DLBCL receiving CHOP regiments who are considered to have an intermediate neutropenia risk (10%‐20%) and no additional risk factors, such as age over 65 years, bone marrow involvement, renal/liver dysfunction, persistent neutropenia, recent surgery, and/or open wounds, do not require prophylactic use of granulocyte colony stimulating factor (G‐CSF) according to NCCN guidelines.^7^ In contrast to the DLBCL group, patients treated with HL receiving ABVD chemotherapy have an overall low neutropenia risk. Although some patients required G‐CSF administrations, the data from healthy donors and stem cell mobilizations indicated that the use of G‐CSF is not associated with an increased risk of thrombotic events.^40^, ^41^ Furthermore, along with the treatment time and achievement of disease control, the risk of developing VTE decreases.

In the presented study, previous VTE/acute myocardial infarction/stroke was identified in univariate analysis as an independent risk factor for VTE events and was associated with an approximately 3.0‐fold increase in the odds for VTE development. Although this finding was not confirmed in multivariate analysis, irrespective of prophylactic aspirin, previous VTE/acute myocardial infarction/stroke appears to be an especially strong risk factor of VTE and could be included in future VTE‐assessment models tailored for patients with lymphoma.

Our study has several strengths. The study population was quite homogenous because we included only consecutive patients treated for newly diagnosed DLBCL and HL within the study period. All patients were managed with the same procedure according to diagnosis and treatment in one hospital so there were no missing data. The study population comprises only a Caucasian population so racial disparities in the risk of thrombosis can be discounted.^42^ Besides, we analyzed only symptomatic VTE events because there was no routine screening for VTE.

There are also limitations. First of all, it is a retrospective analysis of data. Secondly, other subpopulations of lymphoma included in the ThroLy score, particularly chronic lymphocytic leukemia/small lymphocytic lymphoma (14.8% of the ThroLy cohort) were not evaluated. Thirdly, because international guidelines apply to the risk of venous thromboembolisms; we decided it was more important to assess only VTE events.

Furthermore, because the highest risk of VTE is at the beginning of treatment, the evaluation of the VTE risk was performed before the initiation of chemotherapy to identify patients at the highest risk of VTE occurrence, similar to the Khorana study.

In conclusion, in the present study based on newly diagnosed DLBCL and HL patients receiving first‐line treatment, the ThroLy score was not a suitably accurate model for the prediction of VTE events in patients at higher risk of VTE because nearly half of VTE events were found in patients with a low‐risk ThroLy score. The question of whether the ThroLy score identifies lymphoma patients at a high risk of VTE development is still open and it should be clarified in further prospective studies on other lymphoma subpopulations. It seems that the different results of individual studies may be related to population differences and different lymphoma grades. Further prospective trials comparing different lymphoma patients with different VTE risks with or without thromboprophylaxis are required in order to establish the best form of antithrombotic prophylaxis to recommend for this group of patients.

## CONFLICT OF INTEREST

The authors have no conflicts of interest to declare.
